# Supplementary material for: Are carbon emissions trading and green financial instruments synergistic? -Comprehensive quantitative research based on content analysis
Source: PLoS One. 2024 Mar 7;19(3):e0298601. doi: 10.1371/journal.pone.0298601 (PMC10919601; doi:10.1371/journal.pone.0298601)
Supplement: S1 Data — (ZIP) [file pone.0298601.s001.zip › Data/地级市Z_F工作报告.zip!/╜¡╦╒/╠⌐╓▌╩╨/└┤╫╘╣1⁄2╓┌║┼í╛┬φ┐╦╩2╛▌═°í┐_2009.docx]

各位代表：

现在，我代表市人民政府向大会作工作报告，请予审议，并请市政协委员和其他列席人员提出意见。

一、2008年工作回顾

过去的一年，我们在中共泰州市委领导下，深入贯彻党的十七大和十七届三中全会精神，全面落实科学发展观，积极应对宏观形势变化，着力克服发展中的各种困难，扎实推进全市经济、政治、文化与社会建设，较好完成了市三届人大一次会议确定的年度目标任务，顺利实现新一届政府工作的良好开局。

经济实力得到新提升。全年预计实现地区生产总值1394亿元，可比增长14%。其中，第一产业增加值109亿元，增长4.5%；第二产业增加值808亿元，增长14.5%；服务业增加值477亿元，增长15.5%。完成财政收入262.3亿元，增长24.1%，其中地方一般预算收入突破百亿元，达到101.1亿元，增长18.6%。继续推进高效农业“5218工程”，全市高效农业面积扩大到147万亩。认真落实各项惠农政策，粮食总产与夏粮单产创历史新高。兴化再获“全国粮食生产先进县标兵”称号。强化农产品质量建设，市场竞争力进一步增强。加快农业产业化进程，全市新增3家省级、1家国家级重点龙头企业。农业利用“三资”达43亿元。进一步扩大农业政策性保险覆盖面，在全省率先对高效设施农业实施保险。扎实抓好绿化造林工作，全市森林覆盖率达16.1%，泰兴成为省达标市。积极应对一系列挑战，工业经济“高开稳进”。预计全市规模以上工业实现销售收入2850亿元、利税268亿元、利润150亿元，分别增长30%、36%和36%；工业一般纳税人开票销售跨上2000亿元台阶，达2200亿元，增长22%。工业开票销售及利税、利润的增幅均在全省名列前茅。医药、机电（船舶）、化工三大产业和“50强”企业加快发展，不锈钢产业、船舶修造产业入选 “全国百佳产业集群”，机电（船舶）产业成为全市首个销售突破千亿级产业，全市销售过亿元的企业418家、过10亿元的企业32家，分别比上年增加72家和8家。支持企业扩大有效投入，预计全年工业技改财务发生数达567亿元，实施过亿元重大技改项目120个。积极实施品牌战略，新增中国驰名商标2个。大力推进节能减排，万元GDP能耗下降5.5%，二氧化硫排放量和化学需氧量分别削减0.51%、1.85%。建筑业保持较快发展，完成总产值802亿元，增长22.8%。江苏一建承建的北方国际传媒中心项目获得 “鲁班奖”。深入推进服务业“833工程”，出台加快市区服务业发展政策意见，启动市区工业企业二三产业分离工作，现代物流、旅游、金融、保险等行业呈现良好发展势头。预计完成社会消费品零售总额395亿元，增长23.3%。高港综合物流园区、兴化戴南不锈钢综合物流中心被列为省级现代服务业集聚区，全市省级集聚区已达6家。市区望海楼、桃园、老街等重点旅游项目建成开放，华侨城一期建设进展顺利，凤城河风景区通过国家AAAA级景区验收，泰山公园成为国家AAA级景区，溱湖风景区全年接待游客250万人次。年末，全市各项存款（本外币）余额1418亿元、贷款余额803亿元，分别比年初增长24.4%和22.7%，贷款增幅列全省第2位。服务业对地方经济发展的贡献进一步加大，实现地方税收增长28.9%，增收贡献率达56.4%。

改革开放实现新突破。继续深化农村综合配套改革，农村集体财务和土地承包管理工作进一步规范。着力推进农村经营体制改革，农村合作经济组织突破800家，当年新增262家。加大金融体制改革和创新力度，姜堰“阳光信贷”经验在全省推广，兴化、高港等市（区）成立农村小额贷款公司，靖江、泰兴农村合作金融机构的法人治理结构改革成效明显。江苏银行泰州分行、江苏长江商业银行相继成立。鼓励和推动全民创业，民营经济活力进一步释放。全市私营企业总数突破3万家，达3.23万家，当年新增5677家；民营经济净增注册资本168.9亿元，纳税总额突破100亿元。富有成效地开展了赴境外经贸洽谈活动，开放型经济发展势头良好。

预计全年注册协议外资18亿美元，实际利用外资跨上10亿美元台阶，达10.5亿美元；完成自营出口48亿美元，增长62%；新增对外承包经营企业3家，新签外经合同额3.6亿美元，完成营业额4.1亿美元。基地建设取得新突破，以泰州医药高新技术产业园区为核心的生物医药产业基地成为江苏唯一入选的“生物产业国家高技术产业基地”，兴化获批省不锈钢特钢制品出口基地。沿江开发有力推进，在建总投资1亿美元或10亿元人民币以上的重大产业项目19个，泰州长江大桥建设进展顺利，泰州电厂一期工程竣工投产。园区建设步伐加快，8个省级开发区注册协议外资、实际利用外资占全市的比重分别达70%和76%。中国医药城建设取得重要进展，美国加州大学QB3等40多家世界知名医药研发机构以及美国默克、台湾东洋等知名企业先后落户，科技大厦、研发大楼以及新药创制、检验检测、中试研究等公共服务平台相继建成，施耐克、复旦张江等30多个项目开工建设，高通量生物芯片、干细胞等国内领先或国际一流的创新成果正在实施产业化。江阴-靖江开发园区建设和发展取得新进展。积极开展项目上争工作，为泰州今后发展夯实基础。泰州海关、国检、海事、边检等口岸查验机构为开放型经济发展作出了新贡献。外事、侨务和港澳台事务工作取得新成绩，成立泰州市旅港同乡会，与新西兰哈特市结成友好城市。

城乡面貌发生新变化。中心城市新一轮总体规划纲要编制完成，土地利用总体规划编制工作顺利推进。加快“十大重点工程”建设，园博园土方工程、泰州大剧院主体工程和人民路立面改造、东风北路北延、鼓楼南路南延等项目基本完成，建工、春兰等老小区整治全面竣工，新建城北污水处理厂及管网铺设完成阶段目标，凤城河风景区三期开工建设；高港港城路东延、王营河路北延等道路建设加快推进，口岸大桥建成通车，雕花楼景区二期竣工。全年市区城建投资达100亿元。推进市区城市管理和执法工作重心、责任下移，数字化城市管理平台投入运行并通过建设部验收。大力开展拆除违法建设专项行动，累计拆除30多万平方米，违法建设蔓延势头得到有效遏制。各辖市加大城市建设投入，城区面貌发生较大变化。靖江加快推进主城区功能提升和滨江新城建设，泰兴新区建设力度进一步加大，兴化城区防洪工程基本完成，姜堰创建国家环保模范城市通过省级验收。顺利实施部分地区行政区划调整工作，促进区域经济社会持续、协调、共同发展。扎实推进新农村建设，全市新建 （改造）农村公路1000公里，改造农村桥梁470座，疏浚农村河道3700万方土；村庄规划实现全覆盖，新增省级康居示范村6个，省级环境优美乡镇2个，市级环境优美乡镇4个、生态村10个，全面小康示范村200个。靖江、高港率先通过全省农村河道疏浚考核验收，靖江被表彰为“全国农田水利基本建设先进市”。继续强化城乡基础设施建设，积极配合苏中机场完成选址，兴泰公路改扩建先导段实现贯通，江海高速公路建设进展顺利，332省道建成通车；市区备用水源投入使用，刘西河、北城河、老通扬运河一期等河道整治疏浚全面完成，口岸船闸改造竣工通航，靖泰界河整治基本完成；500千伏泰三线等一批重点电力设施建成投运，邮政、通信等基础设施建设取得新的进展。预计全社会固定资产投资完成900亿元，增长27.8%。

社会发展迈出新步伐。继续实施“科教兴市”战略，建设创新型城市。新获批国家创新基金项目18项，扬子江药业集团被命名为全国首批“创新型企业”；新认定企业博士后工作站3家，省级以上工程、技术中心11家，其中国家级技术中心1家，新增国家科技进步奖2项；企业院校行活动成效明显，新增产学研合作项目150项，中科院泰州中心新入驻4家研发分中心；强化知识产权保护工作，新增专利1200件；组织实施国家火炬、星火计划项目46项，新认定国家级高新技术企业35家、高新技术产品116个，新增省级现代农业科技园区2个。成功组织“百名海外博士江苏行泰州洽谈会”等活动，全年引进各类人才1.5万人。区域教育现代化全面推进，义务教育阶段“两免一补”政策实现全覆盖，入学率和巩固率分别达到100%、99%；高中阶段教育教学质量稳步提升，高考本科万人进线率继续位居全省前列；高等教育本科在校生已达1.5万人。加强文化基础设施建设，市区新建市民文化广场10个，学政试院修缮、梅纪馆改造竣工并对外开放。五巷、涵西古街区保护规划通过省专家组评审。传统木船制造技艺等4个项目入选“国家级非物质文化遗产名录”。成功举办首届梅兰芳艺术节。市区数字电视整体平移工作顺利推进，全市有线电视通组（自然村）率100%。健全公共卫生服务体系，城市社区卫生服务覆盖率达90％以上，建成省级卫生镇6个、卫生村43个和市级农村示范社区卫生服务中心15个。及时、有效处置奶制品事件，“放心消费城市”创建活动扎实开展。成功举办市第三届运动会。人口出生率继续稳定在较低水平，计划生育率达99.7%。严格落实建设项目用地预审制、环保前置审批责任制，耕地节约利用和环境保护工作得到进一步加强。我市被表彰为全国第二次农业普查工作先进市。史志档案、社会科学、新闻、统计、物价、气象、无线电管理、防震和民族宗教、妇女儿童、红十字、残疾人等工作继续加强，质监、药监、工商管理为地方经济发展作出了积极贡献，驻泰部队的全面建设、民兵预备役、双拥工作和人民防空事业取得新成绩。

人民生活有了新改善。扎实推进改善民生十大工程，认真办好十件实事，一批事关群众生活的大事难事得到了妥善解决。预计城镇居民人均可支配收入17200元，农民人均纯收入7350元，分别增长15％和13.6％。抓好全国统筹城乡就业试点工作，出台创业带动就业、支持返乡农民创业、动态消除“双零家庭”工作意见，在全国率先推行高校毕业生“就业服务卡”，全年净增城镇就业人员5.3万人，失业人员再就业3.1万人，城镇登记失业率3.15%。在全省率先出台城乡居民社会基本养老保险办法，继续强化社会保险扩面征缴，五大保险新增参保人数48万人。农村社会养老保险新增参保人数20.7万人，已有2.35万农民领取养老金。农村新型合作医疗参保率达98.7%，政府性补助医保资金2.95亿元，受益农民近100万人。开展“劳动保障监察执法年”活动，切实维护劳动者合法权益。重视社会困难群体的生活安排，及时发放物价生活补贴，全面提高城乡居民最低生活保障标准、五保老人供养标准、残疾军人和老复员军人补助标准，对1.55万名无固定收入重残人员、5800名老复员军人遗孀发放生活救助金。进一步完善社会救助体系，建立市区困难群众临时救助制度。兴化成立全省首家社会救助服务中心。建成市儿童福利院。加强住房保障工作，市区符合保障政策的低收入家庭实现应保尽保，廉租房制度惠及573户困难家庭。社区试行基础药物“零差价”，惠民义诊累计减免医疗费用1580万元。优先发展城市公共交通，市区更新公交车辆70辆，新辟线路5条，新建站台12对，新增里程120公里。总投资4.5亿元，管线长达60公里，惠及泰兴、姜堰、海陵、高港等四个市（区）的区域供水工程加快推进，已成功向泰兴水厂供上长江水，泰州市三水厂改扩建和向姜堰供清水干管铺设即将竣工。实施农村饮水安全工程，新增镇村以下受益人口39.5万人。抓好扶贫帮困工作，全市5万贫困人口实现脱贫。大力倡导社会文明关爱，组织第二届“感动泰州”十大人物、精神文明新人新事等评选活动，定期举办“百姓议事园”、“百姓大学堂”。创建全国文明城市工作先进市通过省级考核。加强“平安泰州”、“法治泰州”建设，创建成全国科技强警示范城市，社会公众安全感认可度达98.6%。成功组织奥运火炬传递活动，圆满完成奥运安保任务。我市连续三年被评为省社会治安安全市，首次被评为省社会治安综合治理先进市。深入推进“五五”普法工作，积极开展民主法治示范村（社区）创建，姜堰创建成全国村务公开民主管理示范市。进一步做好信访工作，完善大调解机制，各类信访案件和矛盾纠纷得到妥善处置，群体性事件比上年下降29.2%。扎实开展“安全生产隐患治理年”等活动，全市安全生产形势基本平稳。

自身建设取得新进展。深入开展“践行科学发展观，我为泰州作贡献”主题教育活动，有效解决制约和影响泰州经济社会发展、人民群众反映强烈的热点难点问题。密切关注经济走势，研究部署对策措施，及时出台促进经济增长的8条政策措施和“三服务”活动实施意见，指导和推进经济平稳较快发展。强化政府目标责任管理，落实重大项目领导挂钩督查制度，“411工程”完成情况良好。启动实施新一轮行政审批制度改革，推进行政许可“两集中、两到位”，建成市民服务中心。修订完善《泰州市人民政府工作规则》，出台《规范行政处罚自由裁量权工作指导意见》、《行政问责暂行办法》，推动行政权力网上公开透明运行。全市电子政务网基本建成。组织开展“建设一流软环境”活动，深入查找和解决机关作风建设中存在的薄弱环节，服务发展的环境有了新改善。认真办好市长公开电话、市长信箱和行风热线，进一步畅通人民群众诉求渠道。注重发挥公共财政作用，对社会事业、改善民生实事的投入力度继续加大。建立健全各类应急预案，完善应急体制和机制，应对突发事件能力不断增强。汶川地震发生后，有力有序组织抗灾救灾和对口支援绵竹市拱星镇的恢复重建，及时派出各类救援队伍，救治灾区转运伤员60人，筹集款物计1.07亿元，2个援建项目开工建设。自觉接受市人大及其常委会的法律监督、工作监督和市政协的民主监督，认真执行人大及其常委会决议，重视和发挥民主党派、工商联、无党派人士和群众团体的作用，切实抓好人大代表建议和政协委员提案的办理，综合满意率达100%。认真落实廉政建设和反腐败工作责任制，执纪执法和纠风治乱工作力度加大，行政效能监察不断强化。加强机关事务管理，保障服务能力进一步提升。

各位代表，过去一年政府各项工作取得的成绩，是在中共泰州市委的正确领导下，在市人大、市政协的支持和监督下，全市人民团结拼搏、克难奋进、开拓创新的结果。在此，我代表市人民政府，向全市各行各业的广大干部群众，向各民主党派、工商联和无党派爱国人士，向中央和省驻泰机构以及驻泰部队指战员、武警官兵和公安干警，向所有关心和支持泰州现代化建设的海内外朋友，表示衷心的感谢！

在充分肯定成绩的同时，我们也清醒地看到，全市经济社会发展仍然存在一些矛盾和问题，主要是：科学发展的能力水平有待进一步提高，转变发展方式、加快结构调整的任务还很艰巨；受国际金融危机影响，经济下行风险有所增大，部分工业企业运营困难；农村基础设施建设仍相对滞后，农民持续增收难度明显加大；中心城市功能还不够完善，城市品位和形象也有待进一步提升；改善民生面临新的问题，城乡就业形势较为严峻，社会困难群体生活有待改善；政府自身建设中还存在一些薄弱环节，机关作风建设尚需进一步加强，等等。对上述问题，我们将予以高度重视，采取切实有效措施，在今后的工作中努力克服和解决。

二、2009年工作任务

各位代表，今年是建国60周年，是贯彻落实十七届三中全会精神的重要之年，是全面完成市第三次党代会确定的目标任务的攻坚之年。做好今年的政府工作，具有十分重要的意义。

今年政府工作的总体要求是：全面贯彻党的十七大、十七届三中全会、中央和省经济工作会议精神，认真落实市委三届五次、六次全会决策部署，以科学发展观统领全局，坚定信心，克难奋进，抢抓机遇，力保增长，进一步提升经济发展水平、推进农村改革发展、完善中心城市功能、加快文化泰州建设、优化区域发展环境、促进社会和谐进步，确保全市经济社会平稳较快发展。

综合考虑国内外经济走势以及诸多不确定因素，今年全市国民经济和社会发展主要调控目标为：地区生产总值可比增长11%；财政收入增长11%，其中地方一般预算收入增长11%；全社会固定资产投资增长20%；社会消费品零售总额增长15%；自营出口额增长10%；实际利用外资12亿美元；城镇居民人均可支配收入增长10%，农民人均纯收入增长9%；居民消费价格涨幅不高于全省平均水平；城镇登记失业率控制在4%以内；万元GDP能耗下降4.4%。

在国际金融危机仍在进一步蔓延、实体经济受到冲击仍在进一步加深、经济下行风险仍在进一步加剧的严峻形势下，今年的政府工作将面临前所未有的困难和挑战。为此，必须着力把握好以下几点：一是全力保增长。始终贯彻发展是硬道理的思想，牢牢扭住经济建设这个中心，抓住国家宏观政策调整机遇，采取更为有力的措施，千方百计保持经济平稳较快发展。二是致力上水平。切实转变经济发展方式，大力推进产业结构调整，注重提升自主创新能力，努力在应对危机中调整优化，在调整优化中转型升级。三是不断增活力。加快推进体制、机制创新和重点领域、关键环节的改革，坚定不移地扩大对外开放，切实增强发展的动力和活力。四是始终重民生。把改善民生作为保增长的出发点和落脚点，进一步加大政府性投入力度，认真办好民生实事，着力解决涉及群众切身利益的突出问题，让发展成果更多地惠及广大群众。

今年将重点做好五个方面的工作：

1、着力转变发展方式，进一步推动经济平稳较快发展。围绕保增长这一首要任务，优化产业结构，促进转型升级，提升发展水平。

保持工业经济稳定增长。加强工业经济运行调节，加大财政和信贷支持，着力在保增长上下功夫。实施分类指导，推动重点行业和重点企业加快发展。认真落实促进医药产业发展政策意见，注重放大机电（船舶）产业优势，进一步拉长精细化工产业链条，做大做强三大优势产业；支持“50强”企业加快发展，实施成长型企业发展计划，不断提高企业竞争力；引导企业抢抓国家政策机遇，扩大有效投入，全年实施过亿元重大技改项目140个以上。把增强创新能力作为转变发展方式、优化产业结构的关键举措，走引进、消化吸收、再创新与自主创新相结合之路。推进新一轮产学研合作，重点在新医药、新能源、新材料等领域开展科技合作和攻关；支持企业建立工程、技术研发中心，积极培育创新型企业；加快构建创新服务平台，抓好中科院泰州中心建设；重视知识产权保护，做好国家知识产权示范城市创建迎检工作。坚持节约优先、环保优先，促进可持续发展。继续推进结构、技术和管理节能，突出抓好重点行业、重点企业的节能降耗工作，深入开展节能减排科技支撑行动，积极推进循环经济试点。加强土地资源保护，促进土地节约、高效使用。

提升服务业发展层次。建立服务业与工业、城市建设协调发展机制，落实好鼓励服务业发展的政策措施，进一步加快生产性服务业、新兴服务业发展。积极引进国内外知名物流企业来泰设立分支机构，抓好泰州医药科技创业园、泰州城北物流园、江苏三江现代物流中心等省级现代服务业集聚区建设。大力发展连锁、代理、配送等现代营销方式，加快建设区域性商贸中心和特色市场。推进“金融三区”建设，强化市外商业银行招引，支持现有金融机构做大做强。实施旅游业发展行动计划，抓好凤城河三期（凤凰岛）、华侨城等重点旅游项目建设，举办中国泰州国际旅游节，承办江苏省第六届园博会。落实好房地产业发展政策，调整住房供应结构，发展二手房市场和住房租赁市场，促进房地产市场健康发展。

推动县域经济和市区经济协调发展。加强和改进对县域经济发展的服务，不断提升县域经济综合竞争力。强化市区一体意识，更加突出发展市区经济。进一步理顺市区行政、财政、城建体制，充分发挥海陵、高港和开发区在市区经济发展中的主体作用。切实加大对海陵、高港和开发区发展的支持、协调和服务力度，促进市区经济加快发展、率先发展。

2、努力抓好“三农”工作，进一步促进农村社会全面发展。

以增加农民收入为核心，发展现代农业，推进农村改革，建设社会主义新农村。

积极发展现代农业。继续实施“5218工程”，全市新增高效农业面积15万亩；扩大畜禽养殖规模，扶持发展一批大中型养殖小区和企业，畜禽规模养殖比重提高5个百分点；加快特色渔业板块建设，新增高效渔业面积6万亩。促进农产品提质升档，放大红膏、九寿堂、苏三零、河横等品牌优势，新认证无公害农产品、绿色食品和有机食品30个。推进农业产业化经营，重点龙头企业销售收入增长15%以上。组织实施农业科技更新工程，示范推广50个农业新品种及高效农业新技术。加强农田水利基本建设，突出抓好农村河道疏浚和机电泵站、圩口闸、中低产田改造。完成林业绿化面积10万亩。完善动植物疫病防控体系，保证农产品质量安全。

有序推进农村改革。编制县乡两级土地利用总体规划及村镇土地规划，建立城乡一体的土地利用规划管理体系；组织实施“万顷良田建设工程”，引导农民向新型社区和城镇集中、农田向农业规划区集中；稳定和完善农村基本经营制度，鼓励农民以多种形式流转土地承包经营权，促进适度规模经营，全年力争流转土地10万亩。开展农村小额贷款试点，探索建立农业投资担保公司运作机制，引导金融机构加大支农力度。健全农业生产经营风险预警体系，积极推广高效设施农业保险，增强农业风险规避能力。大力发展农村三大合作组织，提高农村资源配置效率。巩固农村税费改革成果，强化对农民负担和农村财务的监管。

加快发展农村公共事业。认真办好农村新“5+1”实事，进一步改善农村生产生活条件。继续实施农村道路通达工程，新建（改造）农村公路800公里。加强农民工技能培训和返乡农民创业培训，全年实用技术培训15万人、创业培训6000人，新转移农村劳动力5万人。抓好村庄环境整治，鼓励使用清洁能源，努力禁止秸秆焚烧，推广秸秆综合利用，整治疏浚农村河道，实施农村户厕改造，加快城乡垃圾处理一体化进程。重视农村饮水安全，再解决34.7万人安全饮水问题。继续推进“十强争先、百村示范、千村小康”工程，着力抓好示范村建设试点工作。完善农村现代流通服务网络，加快农村社区服务中心建设。强化脱贫攻坚工作，新增农村脱贫人口5.7万人。

3、大力推进对外开放，进一步增强经济发展动力。辩证、准确把握国际国内新形势，把扩大对外开放作为保增长的强大动力，努力实现开放型经济快速发展。

加大招商引资力度。抢抓全球产业重新布局和调整的新机遇，精心组织招商活动。在招引欧美资本的同时，加大对台招商力度，努力让泰州成为新兴台商投资密集区。注重提升引资质量，致力引进高端产业、高端技术、高端人才，引进对结构优化升级起关键和引领作用的重大产业项目。强化以就业为导向的招商引资，引进一批符合生态环保要求的劳动密集型企业。引导重点企业推进国际合作，通过战略性并购重组实现高位嫁接。组建泰州外商投资促进中心，开辟重大外资项目“绿色通道”。举办好科技经贸洽谈会。全年新批超千万美元项目55个以上，力争引进超亿美元项目2至3个。

加快沿江开发步伐。修编沿江开发总体规划，进一步优化沿江产业布局，强化对项目选址的跟踪管理，确保科学、合理、集约开发。按照深水深用、浅水浅用的原则，抓好岸线资源清理整顿，提高岸线利用水平。整合港口资源，高起点规划建设泰州港。扎实抓好沿江重大项目建设，加快中海油沥青及润滑油、安泰动力、新浦苯乙烯等在建重点项目实施进度，力争全年竣工投产1亿美元或10亿元以上项目5个。抢抓泰州长江大桥建设机遇，推进沿江地区与苏南的联动开发。

促进园区转型升级。坚持“产业兴区”发展战略，进一步完善各省级开发区功能分区规划，科学定位园区产业发展方向。抓住国家和省重点扶持的机遇，继续举全市之力推进中国医药城建设，启动建设会展交易区、综合配套区，加快建设疫苗等五大产业基地，进一步完善研发区功能，力争早日获批国家高新区。积极落实赋予市经济开发区的市级管理权限，理顺市开发区与市区其他园区的关系，实现资源共享、优势互补。引导和支持各园区进一步完善金融、科技、物流等生产配套服务，努力降低商务成本。大力推进项目“飞地”开发，促进优势资源向重点开发园区集聚。严格执行项目投资强度标准，强化对园区土地利用率、能源利用率及投入产出率的考核。

抓好外贸、外经工作。用好“国家科技兴贸出口创新基地”平台，加快出口农产品示范区建设，扩大高新技术产品、机电产品和绿色农产品出口。引导出口企业强化品牌建设和争创工作，新增国家、省重点培育的出口名牌2至3个。积极申报国家船舶出口基地。落实出口退税新政策，进一步改善外贸出口环境。加快“走出去”步伐，鼓励企业积极发展境外加工贸易，规范发展对外工程承包和劳务合作。

4、强力推进城市建设，进一步提升中心城市首位度。按照“把泰州建设得更美、更精、更适宜人居和创业”的要求，紧紧围绕“三年再来一个大变化”的目标，掀起新一轮中心城市建设高潮。

加大中心城市建设力度。坚持“市区一体、联动推进”的思路，以“十大重点工程”为抓手，加快中心城市建设。突出抓好“四区”建设，即大力推进以扬桥东北侧、三森街区等为重点的城北旧城区的改造建设，五巷、涵西古街区的改造保护，凤城河风景区的整治建设，周山河街区的有序开发。继续抓好城市功能完善和提升，完成园博园各项工程，建成泰州大剧院，开工建设泰州博物馆、泰州图书馆（美术馆）；启动引江河大道建设，实施泰九路、南通路和扬子江路南延、春港路东延等工程；扩建城南污水处理厂，新建垃圾焚烧发电厂。认真抓好城市绿化，规划建设一批街头小游园，全面开展补绿、增绿、扩绿工作。引导各辖市进一步加快城区建设步伐，推动黄桥、戴南等重点镇向小城市方向发展。

加强城市规划和管理。完成城市总体规划和历史文化名城保护规划编制，启动各专项规划的修编。进一步完善城市管理体制，健全城市管理问责机制，提高城市管理综合执法效能。推进数字城市地理空间框架建设试点，深化数字化城市管理工作。实施无物管小区集中整治，落实长效管理措施。扎实抓好建设环境、交通环境和市容环境的综合整治，巩固拆除违法建设专项行动成果。继续推进国家园林城市创建工作，力争创建成为江苏省节水型城市。

加快重大基础设施建设。做好泰州长江大桥建设、宁启铁路复线及电气化改造的各项配套工作，规划建设泰州港永安核心港区，积极配合苏中机场做好开工准备。启动大桥北接线，实施328国道泰姜段改线，续建江海高速公路，建成兴泰公路扩建段。继续推进城市防洪东北片封闭工程，加快周山河整治、老通扬运河整治、泰东河拓浚、里下河洼地治理等重点水利工程建设，确保区域供水工程竣工。实施220千伏洋桥输变电、220千伏药城输变电项目，年内基本建成投运。开工建设京泰汽车客运站，建成火车站公交停车场。

5、致力改善和保障民生，进一步加强社会建设和管理。认真贯彻“统筹发展、兼顾利益、重在建设”的方针，高度重视社会建设，切实加强社会管理，努力构建和谐社会。

突出抓好就业和社会保障工作。继续实施创业带动就业行动，强化创业培训指导、贷款担保和政策支持，推动民众自主创业。大力发展民营经济，全年净增注册资本110亿元以上。积极推进城乡统筹就业，健全公共就业服务体系，净增城镇就业人员3.8万人。完善面向困难群众的就业援助制度，积极开发公益性岗位，优先安置城乡就业困难人员就业，确保动态消除零就业家庭。认真执行城乡居民社会基本养老保险办法，继续做到“两个确保”。提高农村新型合作医疗保障水平，努力实现保障全覆盖；抓好全国城镇居民基本医疗保险试点，着力解决农民工、困难企业退休人员和职工参保问题。强化劳保监察工作，维护企业职工和进城务工农民的合法权益。为中低收入职工建立住房公积金，市区新建经济适用房10万平方米，落实租售并举保障房源350套。继续做好扶贫帮困工作，对城镇无保障老年居民发放生活补贴，为特困人员提供社会基本养老保险和医疗救助，建立低保边缘人群重病、灾害临时生活救助制度。加快示范性养老机构建设步伐，大力扶持民办养老机构发展。开设市老年活动中心，开工建设市老年公寓。积极发展社会福利和各项慈善事业。

繁荣各项社会事业。坚持教育优先，促进义务教育均衡发展，优化高中阶段教育资源结构，大力发展职业教育。靖江、泰兴、高港力争通过区域教育现代化省级验收。加快泰州大学筹建步伐。加大人才培养引进力度，实施创新型企业人才培训“521工程”，力争引进省级高层次创新创业人才10名以上。整合医疗卫生资源，优化医疗卫生机构布局，启动市人民医院二期建设。加强社区卫生服务体系建设，逐步实现“小病在社区、大病到医院、康复回社区”就医格局。强化疾病预防控制、卫生监督、妇幼卫生保健，改进卫生应急管理，提高公共卫生服务能力。大力发展城市公交，进一步改善市区公交状况。继续推进市区有线电视数字化整体平移，完成泰州中波台迁址新建。深入实施全民健身计划纲要，举办第五届全民健身节，承办好全国男排联赛泰州赛区比赛，备战省第十七届运动会。稳定低生育水平，重视出生人口性别比例偏高和人口老龄化问题。抓好第二次全国经济普查工作。积极发展史志档案、社会科学、新闻、统计、气象、无线电管理、防震等事业，认真做好民族宗教、妇女儿童、残疾人等工作。

加快建设文化泰州。实施2009-2010年“文化泰州建设行动计划”，启动十大先进文化建设工程、发展十大重点文化产业、规划建设十大公共文化设施，推动文化大发展大繁荣。强化历史文化遗存保护，实施名地、名街、名居、名遗工程，积极申报“国家历史文化名城”。建立健全文化艺术创作奖励制度和重点文艺作品资助办法，鼓励文艺创作，推进文艺创新。加强特色文化街区、文化专业市场建设，促进文化产业发展。加快建设乡镇（街道）文化中心和村（社区）文化室，深入开展送书、送戏、送电影到农村活动，推进城市文化下乡、农村文化进城。整合相关文化节庆资源，打造具有泰州特色、影响广泛的文化品牌。完善文化人才选用和激励机制，促进优秀文化人才脱颖而出。

加强精神文明建设。贯彻落实《公民道德建设实施纲要》，加强社会公德、职业道德、家庭美德和未成年人思想道德建设。放大“百姓大学堂”、“百姓大舞台”、“百姓阳光屋”等系列活动品牌效应，发挥“感动泰州十大人物”、“十佳勤政廉政好干部”等先进典型示范作用，着力提升全民素养。在各行各业开展诚信创建活动，培养和树立诚实守信的良好风尚。贯彻落实《公共机构节能条例》，开展节约型机关、节约型单位创建活动。继续加强国防教育、国防动员、后备力量建设和兵员征集工作，组织好海军诞生60周年纪念活动。

做好维护社会稳定工作。继续推进平安泰州、法治泰州建设。深入开展“双排查”活动，完善大信访、大调解工作机制，进一步强化应急管理，积极防范和妥善处置各类矛盾纠纷和群体性事件。坚持“四民主、两公开”，推进农村基层民主法治建设。继续抓好“五五”普法，积极发展法律援助事业。强化流动人口管理、社区矫正、刑释解教人员安置帮教工作，开展无邪教、无毒品、无犯罪、无事故、无非正常上访“五无”村（社区）创建活动，促进基层和谐稳定。建立健全社会治安综合治理组织网络和维稳预警制度，进一步完善“大防控”体系。强化安全生产监督检查，防止重、特大事故发生。实施食品、药品放心工程，保障人民群众生命健康安全。

三、加强政府自身建设

各位代表，科学应对当前宏观经济形势，全面完成今年各项目标任务，对政府工作提出了更高的要求。我们将在能力建设、作风建设、廉政建设上下功夫，切实肩负起建设和谐美好新泰州的重任。

1、加强能力建设，提升施政水平。准确把握发展形势，抢抓积极的财政政策和适度宽松的货币政策机遇，扎实推进一批基础设施、产业和民生类重大项目，努力实现扩大投资规模与优化经济结构并重、民生改善和经济发展双赢；高度关注经济社会发展中的突出矛盾，切实加强应急处置和公共安全保障，不断提升驾驭全局能力。健全重大问题集体决策和专家咨询制度，完善社会公示和听证制度，注重发挥报刊、网络等大众传媒的作用，在决策的全过程坚持问政于民、问需于民、问计于民，不断提升科学决策能力。强化对重点经济指标、重点企业发展和重点工程建设进展情况的监测，着力完善产业发展、招商引资、城市建设等重点工作推进机制，不断提升组织推进能力。扎实开展学习型机关、学习型班子、学习型干部争创活动，引导全体公务员博求新知，增长才干；深入研究涉及全局、关乎长远的重大问题，进一步增强政府工作的系统性和前瞻性。

2、加强作风建设，狠抓工作落实。把机关作风建设作为优化发展环境、推动工作落实的重要抓手，不断开创各项工作新局面。坚持真抓实干，把主要时间和精力放在重点工作、重大项目和为民办实事上，敢于直面矛盾、顶真碰硬，确保各项政策措施落实到位、工作一抓到底；深入开展“三服务”活动，进一步优化投资环境；加强政府执行力建设，健全抓落实的工作机制，强化效能建设和行政问责。坚持勤政为民，继续为市区人民办十件实事，认真办好市长公开电话、市长信箱和行风热线，组织开展领导干部下访，真心诚意为群众办实事、解难事、做好事，努力追求没有水分、群众满意、不留后患的政绩。坚持依法行政，自觉接受市人大及其常委会的监督，支持人民政协履行职责，主动听取各民主党派、工商联、无党派人士以及群众团体的意见和建议，认真办理人大代表建议和政协委员提案；深入贯彻《国务院关于加强市县政府依法行政的决定》，积极推行行政复议委员会试点工作，努力提高行政首长出庭应诉率；认真落实行政执法责任制和执法过错责任追究制，进一步规范行政执法行为；扎实推进行政权力网上公开透明运行，保障公众的知情权和监督权。

3、加强廉政建设，树立良好形象。坚持教育、制度、监督并重，认真落实廉政建设责任制，规范政府工作人员的从政行为。进一步加强廉政文化建设，引导公务员强化廉洁从政意识，筑牢拒腐防变思想道德防线。着力规范公共资源交易行为，完善土地招拍挂、政府采购、工程建设招投标等制度，加大对重点领域、重大工程、重要环节的审计和监察力度，努力从源头上预防和治理腐败。加强行政权力运行监督，严肃查处滥用人事权、审批权等违纪违法案件。深化纠风和专项治理，着力解决群众反映强烈的突出问题。牢固树立过“紧日子”的思想，严控行政开支，降低公务成本，不新建办公用房、不更新购置车辆、不允许公费旅游，为全社会厉行节约、共克时艰做好表率。

各位代表，新的形势催人奋进，新的事业任重道远。让我们紧密团结在以胡锦涛同志为总书记的党中央周围，全面贯彻落实科学发展观，在中共泰州市委的领导下，紧紧依靠全市人民，解放思想、开拓创新，抢抓机遇、克难奋进，为完成全年各项目标任务，加快建设更高水平小康社会进程而努力奋斗！
